# A scale for assessing the health of children aged 3–6 years in China: development and validation of the TCM constitution scale (3–6 years)

**DOI:** 10.3389/fpubh.2025.1690212

**Published:** 2025-11-10

**Authors:** Wenle Li, Siying Dong, Zhuqing Li, Shunqi Chen, Yuyang Cai, Ming-hua Bai, Jia-Xu Chen, Ji Wang

**Affiliations:** 1 School of Chinese Medicine, Beijing University of Chinese Medicine, Beijing, China; 2 School of Traditional Chinese Medicine, Anhui University of Traditional Chinese Medicine, Hefei, China; 3 National Institute of TCM Constitution and Preventive Medicine, Beijing University of Chinese Medicine, Beijing, China; 4 School of Chinese Medicine, Nanjing University of Chinese Medicine, Nanjing, Jiangsu, China; 5 Institute of Basic Theory for Chinese Medicine, China Academy of Chinese Medical Sciences, Beijing, China

**Keywords:** constitution in Chinese medicine scale, body constitution, 3-6 year, psychometric property evaluation, diagnostic validity

## Abstract

**Background:**

Based on the Chinese Medicine constitution, this study developed a questionnaire specifically tailored for preschool children.

**Methods:**

First, a TCM constitution scale for children aged 3–6 years was developed using face-to-face interviews, Delphi expert consultation, and Classical Test Theory (CTT). The Delphi method was conducted via email, and five CTT indicators were selected: decision value–critical ratio, dispersion trend, item–total correlation, internal consistency, and factor loading. Second, the psychometric properties of the scale were assessed, including reliability (internal consistency, test–retest reliability, and split-half reliability) and content validity. Exploratory factor analysis (EFA) was used to evaluate the structural validity of the items.

**Results:**

The CCMQ 2.0 consisted of 47 items selected from the original 49 items of version 1.0. Exploratory factor analysis yielded a Kaiser–Meyer–Olkin (KMO) value of 0.886, and Bartlett’s test of sphericity was significant (*χ*^2^ = 5308.679, *p* < 0.001), indicating that the data were suitable for factor analysis. Nine common factors were extracted, accounting for a cumulative variance contribution of 52.853%. The internal consistency of the CCMQ 2.0 was high, with a Cronbach’s *α* of 0.924, and the test–retest reliability was satisfactory, with an intraclass correlation coefficient (ICC) of 0.86. Overall, the scale showed promising preliminary psychometric properties, although further validation is needed.

**Conclusion:**

This study developed and preliminarily evaluated a 47-item Traditional Chinese Medicine (TCM) constitution scale for assessing the physical and mental health of children aged 3 to 6 years. The scale demonstrated good reliability and validity, providing a promising alternative tool for large-scale pediatric health assessments.

## 1 Introduction

The physical and mental development of children stems from parental genetic inheritance and postnatal nurturing. There is a reciprocal interaction between physical health and emotional states/personality traits. During the preschool period, characterized by social adaptation, children experience alterations in dietary patterns and social environments that differ from their previous familial contexts, potentially leading to developmental shifts in their psychosomatic constitution (1–3). It is imperative for parents to understand preschool children’s developmental status in both physical and psychological domains to facilitate predictive assessments of future growth parameters (height/weight), personality development, and disease susceptibility, thereby enabling proactive prevention of potential developmental challenges (3). Therefore, a scale that integrates four evaluation dimensions—physiological, psychological, natural, and social adaptation—and pathological tendencies is necessary for investigating the physical and mental health development of preschool children.

The constitutional theory of Traditional Chinese medicine has established a new concept of health, which proposes that health refers to the good physical and mental adaptation to the natural and social environment in the whole life cycle, which is exactly what mental and physical health needs now (4). It reflects the current and future individual health trends in four aspects, including physical differences, life processes, psychological conditions, and adaptability to natural and social environments (5). On this basis, nine kinds of BC types are developed: Balance constitution (BC), Qi-deficiency constitution (QDC), Yang-deficiency constitution (YaDC), Yin-deficiency constitution (YiDC), Phlegm-dampness constitution (PDC), Dampness-heat constitution (DHC), Blood-stasis constitution (BSC), Qi-stagnation constitution (QSC), and Special constitution (SC). Each constitution has its representative subjective reaction, objective symptoms, and personality characteristics. The constitution is formed by innate heredity and acquired knowledge. The different constitutions of individuals show some differences in response and adaptability to external stimuli in physiological states, as well as in susceptibility to certain pathogenic factors and the tendency for disease development during pathogenesis (6).

In order to differentiate body constitution, Professor Wang Qi’s research group has developed the Constitution in Chinese Medicine Questionnaire (CCMQ) to assess nine types of constitution and provide guidance on managing body and mental health for people with different constitutions. The psychometric properties of CCMQ have been confirmed (7). At the same time, health management based on constitution identification has been promoted and is well applied in China. Different versions of CCMQ for cross-cultural adaptation have also been promoted and applied in multiple countries, such as the UK and South Korea (8–10). Because the differentiation method of the scale is simple and easy, and it does not harm children, many pediatric experts and educational institutions hope that a children’s version of the CCMQ will be developed (11). However, part of the CCMQ is not applicable to children who have not yet developed secondary sexual characteristics. Therefore, Professor Wang Qi’s research group began to develop scales for different age groups (12). The scales for ages 7–14 and 0–3 have been developed, and psychometric properties have been confirmed (13, 14). Based on the previous study of the research group, the expert experience of preschool children’s physical status was paid more attention than before, and in combination with CTT, the version of the scale for children aged 3–6 years was developed to provide a simple and effective tool for constitution differentiation in early childhood.

## 2 Materials and methods

**Figure 1:**
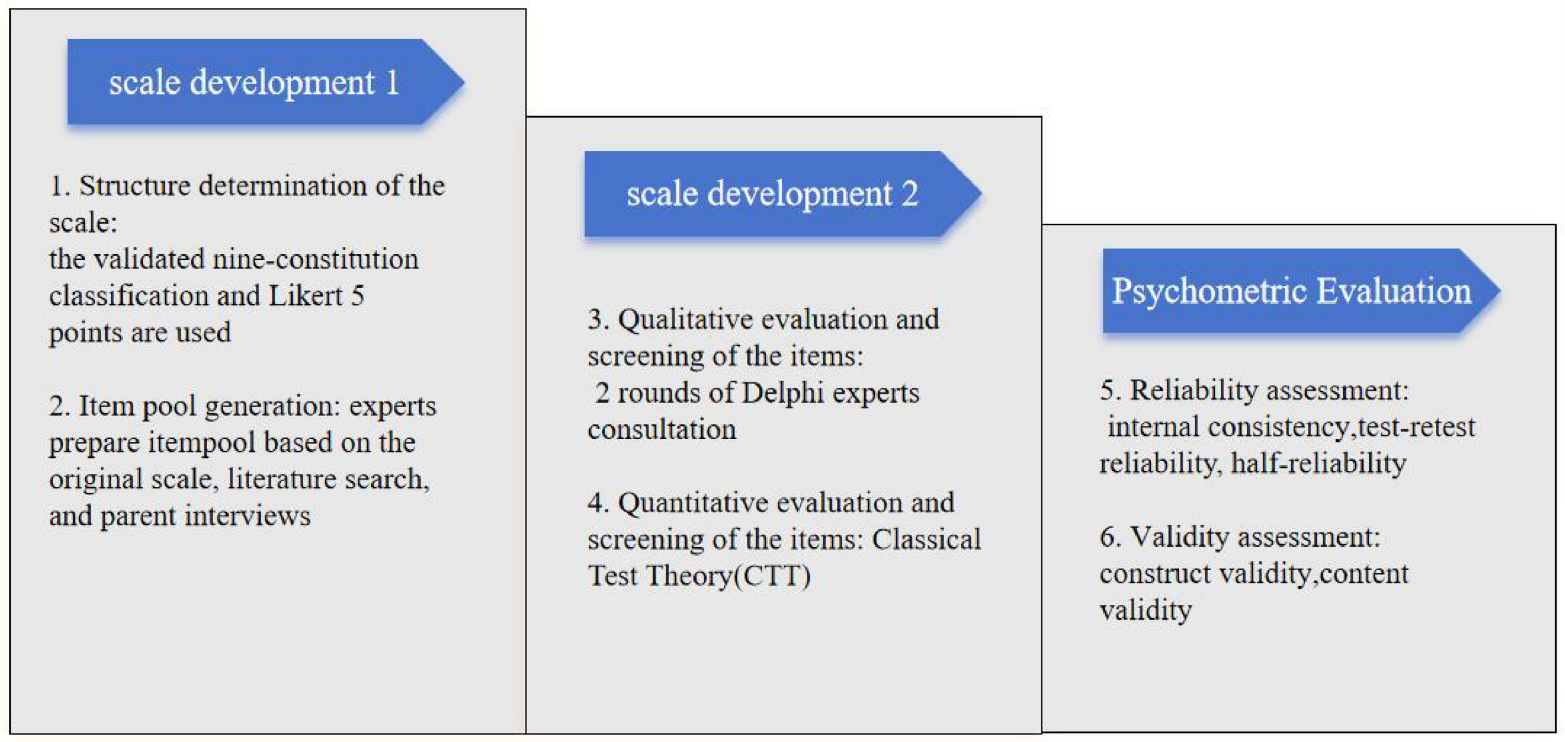
Research process.

This study was divided into scale development and evaluation. The development of CCMQ was an iterative and collaborative process involving item generation, item refinement, item finalization, and scale validation. As a pragmatic process, the study flow is shown in Figure 1. The study was approved by the Ethics Committee of Beijing University of Chinese Medicine (2021BZYLL0102). Preliminary validation has been completed.

### 2.1 Scale development

To develop the items for the 3–6 years version of the TCM constitution, a research group was initially established, consisting of a core group, an expert group, and a survey group. The core group included eight TCM constitution experts, scale research experts, and principal investigators who were responsible for quality control at all stages of the process.

#### 2.1.1 Item generation

This stage aimed to determine the scale’s dimensions and establish an item pool. The scale’s dimensions use nine constitution classification methods, a measurement system with validated psychometric properties (15). The initial draft of the scale was carefully developed by an expert group, drawing on previous research, insights from comprehensive literature reviews and parent interviews, and clinical experience. This also marks the first step in our modified Delphi method. The following is an introduction to each step of our article.

Given the underdeveloped literacy and verbal expression skills of preschool children, parental input should serve as a crucial reference for optimizing item wording in childhood assessment scales. Although a questionnaire for children aged 1–6 years was previously developed, two primary limitations were identified in this version: an excessive number of items and ambiguities in item wording. This existing scale was therefore used as the foundation for conducting parental interviews in the current study. Through face-to-face interviews with 20 parents using the preliminary scale, we systematically compiled and analyzed the findings to formulate reference recommendations, which were subsequently submitted to expert reviewers.

In the literature review, search strategies incorporating keywords such as “preschool,” “children,” “3–6,” “child,” “scales,” “TCM constitution,” and “constitution” were employed across medical literature databases, including CNKI, Wanfang, VIP, PubMed, and Web of Science. The literature search was conducted up to December 2021. Studies were screened and categorized into three thematic categories: (1) theory exploration of children’s constitution, (2) application exploration of constitution scales based on diverse classification methods in children, and (3) relationships between children’s constitution and associated diseases. During this phase, our analysis revealed significant associations between constitution and several age-specific recurrent diseases or single symptoms (such as dental caries, nocturnal enuresis, and rickets). These constitution-related symptoms were subsequently incorporated into the item pool for scale development.

The integration of literature review and interview findings with structured expert discussions serves as an effective preparatory measure to streamline the Delphi expert consultation process (16). Recognizing the inherent disciplinary boundaries of experts from diverse fields, parental feedback, and synthesized literature data were systematically organized and presented as contextual reference materials during expert deliberations. Ultimately, through structured expert brainstorming sessions, we developed an item pool comprising 51 items (as shown in Column 2 of Table 1), with no further elaboration on individual results provided separately.

**Table 1:** Delphi results.

Scale	Item	Round 1						Outcome	Round 2						Outcome
		Importance^a^			Relevance^b^				Importance^a^			Relevance^b^			
		Mean score	Coefficient of variation	Full score ratio	Mean score	Coefficient of variation	Full score ratio		Mean score	Coefficient of variation	Full score ratio	Mean score	Coefficient of variation	Full score ratio	
BC	(1) Is your child always energetic?	4.71	0.12	0.76	4.81	0.11	0.86	Retained without changes	4.86	0.07	0.86	4.9	0.06	0.9	Retained
BC	(2) Does your child easily get sick after changing seasons?	4.52	0.15	0.62	4.52	0.17	0.67	Retained without changes	4.76	0.11	0.81	4.67	0.12	0.71	Retained
BC	(3) Does your child usually fall asleep easily?	4.29	0.22	0.57	4.43	0.17	0.57	Retained with minor changes (3) Does your child usually have difficulty falling asleep and/or easily wake up during sleep?	4.62	0.12	0.67	4.67	0.12	0.71	Retained
BC	(4) Is your child easy to wake up in his sleep?	4	0.31#	0.48	3.95	0.33#	0.48	Deleted	/	/	/	/	/	/	/
BC	/	/	/	/	/	/	/	Newly added (4) Does your child have a normal stool?	4.67	0.12	0.71	4.48	0.18	0.62	Retained
BC	(5) △ Is your child prone to a lack of energy?	3.95	0.35#	0.52	3.57	0.41#	0.38	Deleted	/	/	/	/	/	/	/
BC	/	/	/	/	/	/	/	Newly added (6) △ Is your child always not interested in eating?	4.67	0.14	0.76	4.57	0.2	0.76	Retained
QDC	(5) Is your child prone to a lack of energy?	4.81	0.08	0.81	4.62	0.17	0.76	Retained without changes	4.95	0.04	0.95	4.95	0.04	0.95	Retained
QDC	(6) Is your child always not interested in eating?	4.48	0.15	0.57	4.52	0.15	0.62	Retained without changes	4.67	0.1	0.67	4.71	0.1	0.71	Retained
QDC	(7) Is your child’s complexion too yellowish and dull?	4.52	0.17	0.67	4.48	0.21	0.67	Retained without changes	4.76	0.09	0.76	4.81	0.08	0.81	Retained
QDC	(8) Are your child’s muscles soft and not firm enough?	4.38	0.2	0.57	4.29	0.23	0.57	Retained without changes	4.43	0.15	0.52	4.43	0.15	0.52	Retained
QDC	(9) Is your child prone to catching a cold?	4.71	0.14	0.81	4.71	0.14	0.81	Retained without changes	5	0	1	4.95	0.04	0.95	Retained
QDC	(10) Is your child always very timid?	3.95	0.28#	0.38	3.95	0.25	0.29	Retained without changes	3.9	0.22	0.29	3.95	0.21	0.29	Retained
QDC	(11) Does your child tend to sweat with a little activity?	4.67	0.16	0.76	4.57	0.2	0.71	Retained without changes	4.62	0.17	0.76	4.62	0.17	0.76	Retained
YaDC	(12) Does your child always have cold hands and feet?	4.76	0.09	0.76	4.71	0.12	0.76	Retained without changes	4.95	0.04	0.95	4.95	0.04	0.95	Retained
YaDC	(13) Is your child often afraid of the cold or afraid of eating (drinking) cold things?	4.62	0.17	0.76	4.62	0.17	0.76	Retained without changes	4.9	0.06	0.9	4.9	0.06	0.9	Retained
YaDC	(14) Is your child prone to diarrhea after catching a cold?	4.76	0.11	0.81	4.71	0.14	0.81	Retained without changes	4.81	0.1	0.86	4.76	0.13	0.86	Retained
YaDC	(15) Does your child often wet the bed?	4.05	0.24	0.38	3.86	0.32#	0.33	Retained without changes	3.9	0.22	0.29	3.86	0.23	0.29	Retained
YaDC	(16) Does your child like drinking water?	3.43#	0.44#	0.33	3.48#	0.4#	0.29	Deleted	/	/	/	/	/	/	/
YaDC	(17) Does your child like hot drinks?	4.48	0.13	0.52	4.33	0.17	0.48	Retained without changes	4.29	0.19	0.48	4.29	0.19	0.48	Retained
YiDC	(18) Does your child frequently experience dry skin and lips?	4.67	0.16	0.76	7.05	1.56#	0.71	Retained without changes	4.9	0.06	0.9	4.86	0.07	0.86	Retained
YiDC	(19) Does your child often have dry or globular stools?	4.67	0.12	0.71	4.62	0.14	0.71	Retained without changes	4.81	0.1	0.86	4.76	0.11	0.81	Retained
YiDC	(20) Is your child impatient and prone to losing his temper?	4.43	0.18	0.62	4.38	0.2	0.57	Retained without changes	4.19	0.19	0.38	4.14	0.21	0.38	Retained
YiDC	(21) Does your child prefer something to drink (compared to eating)?	4	0.27#	0.38	3.81	0.33#	0.38	Retained, experts will discuss this item separately	/	/	/	/	/	/	Retained,(21) Does your child have difficulty swallowing or feel uncomfortable when eating dry food?
YiDC	(22) Is your child easily restless or hyperactive?	4.24	0.25	0.48	4.14	0.29#	0.52	Retained without changes	4	0.23	0.29	4	0.23	0.29	Retained
YiDC	(23) Does your child sweat easily when sleeping?	4.71	0.1	0.71	4.62	0.13	0.67	Retained without changes	4.71	0.1	0.71	4.71	0.1	0.71	Retained
PDC	(24) Is your child prone to burnout and not like sports?	4.62	0.14	0.71	4.33	0.21	0.57	Retained without changes	4.57	0.14	0.67	4.57	0.14	0.67	Retained
PDC	(25) Is your child’s belly loose and fat?	4.43	0.18	0.57	4.43	0.18	0.57	Retained without changes	4.52	0.15	0.62	4.48	0.15	0.57	Retained
PDC	(26) Does your child like to eat sweet or greasy things?	4.76	0.11	0.81	4.67	0.14	0.76	Retained without changes	4.86	0.07	0.86	4.76	0.13	0.86	Retained
PDC	(27) Is your child’s stool loose/unformed?	4.57	0.13	0.62	4.38	0.22	0.62	Retained without changes	4.52	0.2	0.67	4.52	0.2	0.67	Retained
PDC	(28) Does your child often have phlegm in his throat?	4.71	0.1	0.71	4.71	0.1	0.71	Retained without changes	4.81	0.08	0.81	4.81	0.08	0.81	Retained
PDC	(29) Is your child’s tongue coating thick?	4.9	0.06	0.9	4.71	0.17	0.86	Retained without changes	4.95	0.04	0.95	4.9	0.06	0.9	Retained
DHC	(30) Do your child’s eyes get red easily or produce discharge?	4.52	0.15	0.62	4.57	0.15	0.67	Retained without changes	4.52	0.13	0.57	4.52	0.15	0.62	Retained
DHC	(31) Does your child often urinate yellow?	4.57	0.15	0.67	4.62	0.13	0.67	Retained without changes	4.76	0.11	0.81	4.71	0.12	0.76	Retained
DHC	(32) Does your child always have bad breath?	4.67	0.12	0.71	4.71	0.12	0.76	Retained without changes	4.86	0.1	0.9	4.86	0.1	0.9	Retained
DHC	(33) Does your child often have perianal eczema or red itching?	4.52	0.19	0.71	4.38	0.2	0.62	Retained without changes	4.67	0.12	0.71	4.57	0.14	0.67	Retained
DHC	(34) Does your child often have wheat grains?	3.9	0.36#	0.52	3.71	0.37#	0.38	Deleted	/	/	/	/	/	/	/
DHC	(35) Are your child’s lips slightly red?	4.33	0.23	0.62	4.29	0.23	0.57	Retained without changes	4.14	0.2	0.38	4.1	0.2	0.33	Retained
BSC	(36) Does your child have purplish-dark nails (fingers/toes)?	4.33	0.23	0.62	4.38	0.22	0.62	Retained without changes	4.48	0.19	0.67	4.57	0.17	0.71	Retained
BSC	(37) Does your child have petechiae on the lips or tongue?	4.52	0.18	0.67	4.43	0.22	0.67	Retained without changes	4.57	0.17	0.71	4.52	0.23	0.76	Retained
BSC	(38) Is your child prone to blue and purple ecchymosis after bumping or fading slowly after occurrence?	4.43	0.21	0.67	4.48	0.2	0.67	Retained without changes	4.43	0.18	0.57	4.52	0.18	0.67	Retained
BSC	(39) Is your child’s skin or neck, or the base of the armpit or thigh dark?	4.14	0.22	0.43	4.29	0.2	0.48	Retained without changes	3.95	0.2	0.24	4.05	0.19	0.29	Retained
QSC	(40) Does your child have a cheerful personality?	4.76	0.09	0.76	4.81	0.14	0.9	Retained without changes	4.9	0.06	0.9	4.9	0.06	0.9	Retained
QSC	(41) Is your child prone to fear or fright?	4.19	0.22	0.48	4.14	0.22	0.43	Retained without changes	4.1	0.22	0.43	4.1	0.22	0.43	Retained
QSC	(42) Does your child always cry when encountering difficulties?	4.48	0.15	0.57	4.52	0.15	0.62	Retained without changes	4.48	0.13	0.52	4.48	0.13	0.52	Retained
QSC	(43) Is your child too sensitive or too concerned about the attitude of others?	4.67	0.12	0.71	4.71	0.12	0.76	Retained without changes	4.81	0.1	0.86	4.86	0.1	0.9	Retained
QSC	(44) Is your child green at the root of his nose (in the middle of his eyebrows)?	3.9	0.36#	0.52	4.1	0.32#	0.52	Retained without changes	3.95	0.25	0.33	4.05	0.23	0.33	Retained
QSC	(45) Is your child prone to being too excited or nervous?	4.71	0.1	0.71	4.71	0.1	0.71	Retained without changes	4.76	0.09	0.76	4.76	0.09	0.76	Retained
SC	(46) Does your child often sneeze in the morning or after catching a cold?	4.86	0.07	0.86	4.9	0.06	0.9	Retained without changes	5	0	1	5	0	1	Retained
SC	(47) Does your child often have a stuffy nose and a runny nose when he is not sick? (Over 2 weeks)	4.71	0.15	0.81	4.57	0.2	0.76	Retained without changes	4.95	0.04	0.95	4.9	0.06	0.9	Retained
SC	(48) Is your child prone to allergies (to medication, food, smell, pollen, or during seasons and climate change)?	4.81	0.08	0.81	4.95	0.04	0.95	Retained without changes	4.95	0.04	0.95	4.95	0.04	0.95	Retained
SC	(49) Does your child often have a skin rash?	4.86	0.07	0.86	4.86	0.07	0.86	Retained without changes	4.95	0.04	0.95	4.95	0.04	0.95	Retained
SC	(50) Does your child like rubbing his nose or scratching his skin?	4.67	0.14	0.76	4.67	0.14	0.76	Retained without changes	4.71	0.12	0.76	4.71	0.12	0.76	Retained
SC	(51) Is your child prone to dark circles under the eyes?	3.95	0.38#	0.57	3.86	0.39#	0.52	Retained without changes	4.1	0.29#	0.52	4.19	0.24	0.52	Retained

BC, Balance constitution (Tizhi); QDC, Qi-deficiency constitution (Tizhi); YaDC, Yang-deficiency constitution (Tizhi); YiDC, Yin-deficiency constitution (Tizhi); PDC, Phlegm-dampness constitution (Tizhi), DHC, Damp-heat constitution (Tizhi), BSC, Blood-stasis constitution (Tizhi), QSC, Qi-stagnation constitution (Tizhi), SC, Special constitution (Tizhi); △, Reverse points item; #, Outlier; ^a^, Delphi experts’ scoring on the importance of items; ^b^, Delphi experts’ scoring on the rationality of items.

#### 2.1.2 Qualitative item screening

This phase employed a Delphi expert survey to further screen and refine scale items using both qualitative and quantitative approaches.

##### 2.1.2.1 Participants

A total of 21 experts were invited from a pre-established expert database and met the following inclusion criteria: 1. holding a bachelor’s degree or higher, with ≥10 years of research experience in their discipline and mid-level or higher professional titles; 2. demonstrated expertise or research background in TCM constitutional theory; 3. the experts’ primary workplace locations were equitably distributed across different administrative regions; 4. high engagement, willingness, and availability to complete two rounds of Delphi consultations; and 5. enough pediatric specialists who can cover comprehensive clinical domains.

Demographic characteristics of the experts are summarized in Table 2. To address the unique demands of pediatric practice, the selection prioritized clinicians with extensive pediatric experience and multidisciplinary expertise, thereby ensuring the authority of the expert panel.

**Table 2:** General information of the Delphi experts (*n* = 21).

Basic information about experts		Values
Sex	Male	6 (28.6%)
	Female	15 (71.4%)
Age (year)	40–50	8 (38.1%)
	51–60	9 (42.9%)
	61–70	4 (19%)
Education	Bachelor Degree	2 (9.5%)
	Master Degree	4 (19%)
	Doctor Degree	15 (71.4%)
Professional title	Intermediate title	1 (4.8%)
	Advanced title	19 (90.5%)
Professional field	Pediatrics of TCM	11 (52.4%)
	Constitution of TCM	5 (23.8%)
	Diagnostics of TCM	1 (4.8%)
	Psychology	1 (4.8%)
	Scale	1 (4.8%)
	Evidence-based medicine	2 (9.5%)
	Nutriology	1 (4.8%)
Work experience (year)	<20	5 (23.8%)
	20–29	4 (19%)
	≥30	12 (57.1%)
Geographical distribution	North China (Beijing, Shanxi)	6 (28.6%)
	Northeast China (Heilongjiang and Jilin)	2 (9.5%)
	East China (Jiangsu and Shanghai)	4 (19%)
	Central China (Henan and Hunan)	2 (9.5%)
	South China (Guangdong)	3 (14.3%)
	Southwest China (Sichuan)	2 (9.5%)
	Northwest China (Xinjiang and Gansu)	2 (9.5%)
Work unit	Comprehensive Hospital	2 (9.5%)
	colleges and universities	3 (14.3%)
	Comprehensive Hospital/colleges and universities	16 (76.2%)

##### 2.1.2.2 Consultation process and statistical analysis

The Delphi expert consultations were conducted via email. The questionnaire solicited expert opinions on key methodological components, including dimensional classification, item scoring methodology, and the temporal design of the scale. Each domain encompassed distinct functional aspects and hierarchical levels, with all items evaluated using a 5-point Likert scale. These were all recognized by the experts in Round 1.

###### 2.1.2.2.1 Computational methodology

Quantitative indices of importance and rationality were operationalized through the following metrics: mean scores, full-score ratio, coefficient of variation (CV), and Kendall’s W coefficient. The core group applied predetermined thresholds for item selection and refinement: a mean score of > 3.50 (moderate-critical threshold), a full-score ratio of > 0.20 (substantial agreement criterion), and a CV of ≤ 0.25 (low dispersion requirement) (17). These analytical procedures ensured methodological rigor in optimizing the scale’s psychometric properties, as detailed in Table 1.

#### 2.1.3 Quantitative item screening

##### 2.1.3.1 Participants and procedures

Between November and December 2022, parents or guardians (including biological parents, grandparents, or other primary caregivers) of children aged 3–6 years were recruited from Beijing, Shijiazhuang, Shandong, and Yunnan to complete an online survey. Inclusion criteria: 1. children aged 3–6 years; 2. caregivers providing long-term care with a comprehensive understanding of the child’s health status; and 3. voluntary participation with signed informed consent. Exclusion criteria: 1. non-eligible caregivers per inclusion standards; 2. families with psychiatric disorders or consciousness-related behavioral impairments; and 3. severe medical conditions precluding survey participation.

Participants were recruited through flyers distributed by universities and local schools. Informed consent was obtained and filled out by WJX (an online questionnaire survey platform). After cleaning the data to remove incomplete or duplicate responses, the final analytical sample comprised 357 participants.

##### 2.1.3.2 Data processing and statistical analysis

Valid responses collected via the WJX platform were organized in Excel and analyzed using SPSS 28.0. By employing online questionnaires, multiple data sources, and age-stratified sampling, this study minimized the potential influence of social bias to the greatest extent possible. However, the possibility of bias cannot be completely ruled out, particularly for subjective items. The items were psychometrically evaluated through the following analytical methods: item discrimination was assessed using the high-low group comparison method (27% extreme scores method) (critical ratio [CR] via extreme score differentiation); item representativeness was determined through item-total correlation coefficients; internal consistency of both the composite scale and subscales was evaluated using Cronbach’s *α* coefficient; item sensitivity was measured via the coefficient of variation (CV) to quantify dispersion trends; and construct contribution was examined through factor loadings in dimensional analyses (17, 18). Items satisfying the four analytical methods were retained. The remaining items were appropriately trimmed as required for the scale construction.

### 2.2 Part 2: psychometric properties analysis

Following the development of the CCMQ for Children, Version 2.0, we preliminarily evaluated its psychometric properties. Participant inclusion and exclusion criteria, data collection protocols, and analytical software (SPSS 28.0) remained consistent with Section 2.1.3.1 and are therefore not reiterated.

Internal consistency was evaluated using Cronbach’s *α* coefficient; test–retest reliability was assessed using the intraclass correlation coefficient (ICC), and split-half reliability was evaluated via the Guttman split-half coefficient; for validity assessment, construct validity was validated through exploratory factor analysis (EFA) (15, 18).

## 3 Results

As described in the Methods section, the research results are presented in two parts: scale development and psychometric property analysis.

### 3.1 Item screening

#### 3.1.1 Delphi method item screening

The expert consultation process involved two rounds, with both achieving a 100% valid response rate for the questionnaires. The experts’ authority coefficients were 0.86 and 0.826, respectively. The Kendall coefficient of concordance (W) for rationality was 0.24 (*p* = 0.00) and 0.282 (*p* = 0.00), while those for importance were 0.21 (*p* = 0.00) and 0.299 (*p* = 0.00). The experts demonstrated high authority and active engagement. Based on their feedback, items were revised and adjusted. After the first round of adjustments, 49 items were retained. In the second round, expert opinions showed strong convergence and coordination. Following the discussion, the number of items remained unchanged, with only minor wording modifications. The outcomes of item adjustments and retention are presented in Table 1.

#### 3.1.2 Results of classical test theory (CTT) screening

Items meeting ≥4 analytical criteria were retained, while others were appropriately revised based on scale construction requirements: High-Low Grouping Method: All items showed statistical significance (*p* < 0.05). Item 16 had *t* = 2.523 (*p* = 0.013), while other items exhibited *t* > 3.0. Item-Total Correlation: Items 1, 4, 15, 16, 24, and 35 had corrected item-total correlations < 0.3, though all correlations were statistically significant (*p* < 0.05). Cronbach’s *α* Coefficient: Items 15 and 16 reduced the α coefficient of the Yang-deficiency constitution subscale. Deletion of items 33 (Damp-heat), 38/42 (Qi-stagnation), and 49 (Special diathesis) caused negligible α fluctuations (Δ*α* < 0.02). Dispersion Trend: CV > 0.25 for all items, indicating items have adequate sensitivity. Factor Loadings: Item 15 loading = 0.173; Item 16 loading = 0.297 (both < 0.40 threshold). After panel review, Item 4 (“Does your child have a normal stool?”) and Item 15 (“Does your child often wet the bed?”) were removed, retaining 47 items (Table 3).

**Table 3:** Results of classical test theory (CTT) screening.

Scale	Item	Criteria value-critical ratio method	Item-total correlation method	Internal consistency method	Discrete tendency method	Factor loading	Outcome
BC	(1) Is your child always energetic?	4.072	0.231^**^	0.418	0.41	0.413	Retained
BC	(2) Does your child easily get sick after changing seasons?	8.981	0.513^**^	0.329	0.29	0.67	Retained
BC	(3) Does your child usually have difficulty falling asleep and/or easily wake up during sleep?	7.69	0.428^**^	0.331	0.41	0.67	Retained
BC	(4) Does your child have a normal stool?	4.027	0.276^**^	0.41	0.45	0.435	Retained
BC	(6) △ Is your child always not interested in eating?	6.824	0.450^**^	0.381	0.42	0.541	Retained
QDC	(5) △ Is your child prone to a lack of energy?	9.321	0.529^**^	0.7	0.39	0.572	Retained
QDC	(6) Is your child always not interested in eating?	6.824	0.450^**^	0.72	0.42	0.651	Retained
QDC	(7) Is your child’s complexion too yellowish and dull?	9.646	0.509^**^	0.69	0.53	0.668	Retained
QDC	(8) Are your child’s muscles soft and not firm enough?	10.788	0.555^**^	0.67	0.58	0.756	Retained
QDC	(9) Is your child prone to catching a cold?	10.33	0.589^**^	0.68	0.37	0.693	Retained
QDC	(10) Is your child always very timid?	10.005	0.506^**^	0.71	0.39	0.592	Retained
QDC	(11) Does your child tend to sweat with a little activity?	6.634	0.438^**^	0.74	0.37	0.443	Retained
YaDC	(12) Does your child always have cold hands and feet?	10.278	0.567^**^	0.388	0.46	0.736	Retained
YaDC	(13) Is your child often afraid of the cold or afraid of eating (drinking) cold things?	9.358	0.458^**^	0.313	0.5	0.811	Retained
YaDC	(14) Is your child prone to diarrhea after catching a cold?	11.17	0.543^**^	0.351	0.43	0.782	Retained
YaDC	(15) Does your child often wet the bed?	3.657	0.181^**^	0.554	0.5	0.173	Deleted
YaDC	(16) Does your child like hot drinks?	2.523	0.140*	0.577	0.41	0.297	Retained
YiDC	(17) Does your child frequently experience dry skin and lips?	8.651	0.485^**^	0.679	0.4	0.56	Retained
YiDC	(18) Does your child often have dry or globular stools?	8.303	0.484^**^	0.659	0.41	0.631	Retained
YiDC	(19) Is your child impatient and prone to losing his temper?	8.773	0.555^**^	0.639	0.35	0.704	Retained
YiDC	(20) Does your child have difficulty swallowing or feel uncomfortable when eating dry food?	9.535	0.535^**^	0.664	0.44	0.622	Retained
YiDC	(21) Is your child easily restless or hyperactive?	9.001	0.531^**^	0.639	0.49	0.695	Retained
YiDC	(22) Does your child sweat easily when sleeping?	7.51	0.490^**^	0.676	0.43	0.585	Retained
PDC	(23) Is your child prone to burnout and not like sports?	7.776	0.443^**^	0.586	0.48	0.583	Retained
PDC	(24) Is your child’s belly loose and fat?	4.289	0.280^**^	0.585	0.54	0.578	Retained
PDC	(25) Does your child like to eat sweet or greasy things?	6.228	0.400^**^	0.606	0.37	0.536	Retained
PDC	(26) Is your child’s stool loose/unformed?	7.541	0.357^**^	0.596	0.45	0.544	Retained
PDC	(27) Does your child often have phlegm in his throat?	12.815	0.586^**^	0.582	0.45	0.613	Retained
PDC	(28) Is your child’s tongue coating thick?	11.811	0.641^**^	0.544	0.41	0.695	Retained
DHC	(29) Do your child’s eyes get red easily or produce discharge?	12.04	0.597^**^	0.55	0.42	0.709	Retained
DHC	(30) Does your child often urinate yellow?	10.746	0.586^**^	0.496	0.37	0.772	Retained
DHC	(31) Does your child always have bad breath?	10.521	0.555^**^	0.561	0.42	0.69	Retained
DHC	(32) Does your child often have perianal eczema or red itching?	5.278	0.345^**^	0.6	0.5	0.542	Retained
DHC	(33) Are your child’s lips slightly red?	6.624	0.368^**^	0.648	0.45	0.455	Retained
BSC	(34) Does your child have purplish-dark nails (fingers/toes)?	6.176	0.339^**^	0.482	0.41	0.726	Retained
BSC	(35) Does your child have petechiae on the lips or tongue?	4.42	0.241^**^	0.506	0.39	0.687	Retained
BSC	(36) Is your child prone to blue and purple ecchymosis after bumping or fading slowly after occurrence?	9.964	0.537^**^	0.545	0.52	0.634	Retained
BSC	(37) Is your child’s skin or neck, or at the base of the armpit or thigh dark?	6.966	0.424^**^	0.445	0.53	0.682	Retained
QSC	(38) Does your child have a cheerful personality?	6.268	0.302^**^	0.745	0.42	0.503	Retained
QSC	(39) Is your child prone to fear or fright?	10.254	0.534^**^	0.695	0.36	0.719	Retained
QSC	(40) Does your child always cry when encountering difficulties?	9.463	0.572^**^	0.681	0.36	0.768	Retained
QSC	(41) Is your child too sensitive or too concerned about the attitude of others?	9.991	0.510^**^	0.672	0.4	0.776	Retained
QSC	(42) Is your child green at the root of his nose (in the middle of his eyebrows)?	9.142	0.499^**^	0.771	0.62	0.407	Retained
QSC	(43) Is your child prone to being too excited or nervous?	11.683	0.620^**^	0.667	0.41	0.784	Retained
SC	(44) Does your child often sneeze in the morning or after catching a cold?	10.824	0.578^**^	0.787	0.45	0.704	Retained
SC	(45) Does your child often have a stuffy nose and a runny nose when he is not sick? (Over 2 weeks)	10.857	0.550^**^	0.778	0.54	0.749	Retained
SC	(46) Is your child prone to allergies (to medication, food, smell, pollen, or during seasons and climate change)?	7.695	0.466^**^	0.76	0.57	0.809	Retained
SC	(47) Does your child often have a skin rash?	7.403	0.405^**^	0.785	0.53	0.718	Retained
SC	(48) Does your child like rubbing his nose or scratching his skin?	9.762	0.521^**^	0.766	0.5	0.782	Retained
SC	(49) Is your child prone to dark circles under the eyes?	11.534	0.547^**^	0.818	0.56	0.544	Retained

BC, Balance constitution (Tizhi); QDC, Qi-deficiency constitution (Tizhi); YaDC, Yang-deficiency constitution (Tizhi); YiDC, Yin-deficiency constitution (Tizhi); PDC, Phlegm-dampness constitution (Tizhi), DHC, Damp-heat constitution (Tizhi), BSC, Blood-stasis constitution (Tizhi), QSC, Qi-stagnation constitution (Tizhi), SC, Special constitution (Tizhi). The value of Criteria value-critical ratio method is the *t*-value of the independent sample *t*-test, the value of Item-total correlation method is the correlation coefficient of item-scale score, the internal consistency method is Cronbach’s alpha coefficient of body constitution scale after deleting items, the value of discrete tendency method is the SD of the mean item score, the factor loading method is the factor loading value of the item in the body constitution scale belonged. *The significance level is 0.05 (*p* < 0.05). **The significance level is 0.01 (*p* < 0.01).

### 3.2 Psychometric evaluation

In this stage, 350 questionnaires were distributed, and 332 completed questionnaires were returned. The scale recovery rate was 94.85%, and the response rate reached 96.98%, indicating a high feasibility of the scale.

#### 3.2.1 Internal consistency

Cronbach’s *α* coefficient for the full scale was 0.924, indicating good internal consistency. The Cronbach’s α coefficients for the subscales ranged from 0.536 to 0.797, suggesting acceptable reliability across all nine subscales.

#### 3.2.2 Split-half reliability

The Spearman-Brown coefficient for the full scale was 0.883, indicating satisfactory split-half reliability. Subscale Spearman-Brown coefficients ranged from 0.479 to 0.772, reflecting moderate split-half reliability for individual subscales.

#### 3.2.3 Test–retest reliability

The test–retest analysis included 49 participants. The intraclass correlation coefficient (ICC) for the full scale was 0.86, with ICC values for the subscales ranging from 0.735 to 0.879.

The results of Sections 3.2.1 to 3.2.3 are comprehensively presented in Table 4.

**Table 4:** Reliability assessment.

Constitution type	Item number	Test–retest reliability coefficient	Spearman Brown coefficient	Cronbach’s alpha coefficient
BC	5	0.847	0.674	0.622
QDC	7	0.879	0.557	0.708
YaDC	4	0.735	0.479	0.536
YiDC	6	0.775	0.655	0.721
PDC	6	0.821	0.618	0.664
DHC	5	0.761	0.629	0.667
BSC	4	0.843	0.538	0.579
QSC	6	0.852	0.657	0.681
ISC	6	0.827	0.772	0.797
Total scale	47	0.86	0.883	0.924

BC, Balance constitution (Tizhi); QDC, Qi-deficiency constitution (Tizhi); YaDC, Yang-deficiency constitution (Tizhi); YiDC, Yin-deficiency constitution (Tizhi); PDC, Phlegm-dampness constitution (Tizhi), DHC, Damp-heat constitution (Tizhi), BSC, Blood-stasis constitution (Tizhi), QSC, Qi-stagnation constitution (Tizhi), SC, Special constitution (Tizhi).

#### 3.2.4 Content validity

The Delphi expert consultation method was employed to validate the rationality and importance of the scale items. Both the concentration and coordination of expert opinions were high. Additionally, the satisfactory test–retest reliability results obtained in later stages further supported the good content validity of the scale.

#### 3.2.5 Construct validity

The KMO measure for the scale was 0.886, and Bartlett’s test of sphericity yielded *χ*^2^ = 5308.679 (*p* < 0.001), confirming the suitability for factor analysis. Under natural extraction conditions, 13 common factors were identified, accounting for 62.156% of the cumulative variance. When specifying nine factors (aligned with the subscale design), the cumulative variance explained was 52.853%, indicating adequate explanatory power for constitution-type classification. Detailed results for the full scale and subscales are provided in Table 5, while rotated factor loadings and distribution patterns are presented in Table 6.

**Table 5:** Total variance explained for the total scale under natural conditions.

Scale	Component	Initial eigenvalues			Extraction sums of squared loadings			Rotation sums of squared loadings		
		Total	% of Variance	Cumulative %	Total	% of Variance	Cumulative %	Total	% of Variance	Cumulative %
Total scale	1	11.104	23.625	23.625	11.104	23.625	23.625	3.245	6.905	6.905
	2	2.503	5.326	28.95	2.503	5.326	28.95	3.221	6.853	13.758
	3	2.371	5.045	33.996	2.371	5.045	33.996	2.79	5.935	19.693
	4	1.893	4.029	38.024	1.893	4.029	38.024	2.556	5.438	25.131
	5	1.623	3.452	41.477	1.623	3.452	41.477	2.438	5.188	30.319
	6	1.449	3.084	44.561	1.449	3.084	44.561	2.43	5.169	35.488
	7	1.386	2.949	47.51	1.386	2.949	47.51	2.369	5.041	40.529
	8	1.283	2.729	50.239	1.283	2.729	50.239	2.314	4.924	45.453
	9	1.229	2.614	52.853	1.229	2.614	52.853	1.729	3.68	49.132
	10	1.167	2.483	55.336	1.167	2.483	55.336	1.602	3.408	52.541
	11	1.114	2.37	57.706	1.114	2.37	57.706	1.532	3.261	55.801
	12	1.058	2.251	59.956	1.058	2.251	59.956	1.524	3.242	59.043
	13	1.038	2.209	62.165	1.038	2.209	62.165	1.468	3.123	62.165
	14	0.973	2.07	64.235						
	15	0.924	1.966	66.201						
	16	0.856	1.821	68.022						
	17	0.842	1.791	69.813						
	18	0.814	1.733	71.546						
	19	0.774	1.648	73.194						
	20	0.736	1.565	74.759						
	21	0.717	1.525	76.284						
	22	0.678	1.443	77.727						
	23	0.66	1.404	79.131						
	24	0.652	1.387	80.517						
	25	0.627	1.334	81.851						
	26	0.588	1.252	83.103						
	27	0.549	1.169	84.271						
	28	0.546	1.161	85.432						

29	0.509	1.084	86.516						

	30	0.497	1.058	87.574						
	31	0.471	1.002	88.576						
	32	0.461	0.981	89.557						
	33	0.452	0.962	90.519						
	34	0.443	0.943	91.462						
	35	0.411	0.875	92.338						
	36	0.397	0.844	93.182						
	37	0.381	0.811	93.993						
	38	0.351	0.746	94.739						
	39	0.346	0.735	95.474						
	40	0.316	0.672	96.146						
	41	0.306	0.652	96.799						
	42	0.291	0.62	97.419						
	43	0.276	0.586	98.005						
	44	0.253	0.538	98.543						
	45	0.236	0.503	99.046						
	46	0.232	0.493	99.539						
47	0.217	0.461	100						
BC	1	2.104	42.071	42.071	2.104	42.071	42.071	1.65	33.003	33.003
	2	1.04	20.805	62.876	1.04	20.805	62.876	1.494	29.873	62.876
	3	0.764	15.273	78.149						
	4	0.648	12.959	91.109						
	5	0.445	8.891	100						
QDC	1	2.654	37.909	37.909	2.654	37.909	37.909	1.959	27.99	27.99
	2	1.206	17.229	55.138	1.206	17.229	55.138	1.9	27.148	55.138
	3	0.772	11.029	66.167						
	4	0.735	10.495	76.662						
	5	0.703	10.036	86.699						
	6	0.551	7.873	94.572						
	7	0.38	5.428	100						
YaDC	1	1.819	45.466	45.466	1.819	45.466	45.466			
	2	0.949	23.724	69.189						
	3	0.67	16.753	85.943						
	4	0.562	14.057	100						
YiDC	1	2.54	42.333	42.333	2.54	42.333	42.333			
	2	0.909	15.155	57.488						
	3	0.749	12.485	69.973						
	4	0.68	11.331	81.304						
	5	0.628	10.465	91.769						
	6	0.494	8.231	100						
PDC	1	2.293	38.209	38.209	2.293	38.209	38.209			
	2	0.924	15.398	53.607						
	3	0.82	13.671	67.278						
	4	0.769	12.818	80.097						
	5	0.626	10.437	90.534						
	6	0.568	9.466	100						
DHC	1	2.196	43.921	43.921	2.196	43.921	43.921			
	2	0.845	16.908	60.829						
	3	0.748	14.95	75.779						
	4	0.656	13.121	88.9						
	5	0.555	11.1	100						
BSC	1	2.031	50.766	50.766	2.031	50.766	50.766			
	2	0.893	22.336	73.101						
	3	0.725	18.114	91.216						
	4	0.351	8.784	100						
QSC	1	2.41	40.175	40.175	2.41	40.175	40.175			
	2	0.968	16.135	56.31						
	3	0.936	15.592	71.902						
	4	0.623	10.389	82.291						
	5	0.564	9.404	91.695						
	6	0.498	8.305	100						
SC	1	3.012	50.204	50.204	3.012	50.204	50.204			
	2	0.831	13.851	64.055						
	3	0.722	12.041	76.096						
	4	0.572	9.526	85.622						
	5	0.472	7.874	93.496						
	6	0.39	6.504	100						

BC, Balance constitution (Tizhi); QDC, Qi-deficiency constitution (Tizhi); YaDC, Yang-deficiency constitution (Tizhi); YiDC, Yin-deficiency constitution (Tizhi); PDC, Phlegm-dampness constitution (Tizhi), DHC, Damp-heat constitution (Tizhi), BSC, Blood-stasis constitution (Tizhi), QSC, Qi-stagnation constitution (Tizhi), SC, Special constitution (Tizhi).

**Table 6:** Rotated component matrix^a^.

Constitution type	Item	Component								
		1	2	3	4	5	6	7	8	9
BC	(1) Is your child always energetic?							0.743		
	(2) Does your child easily get sick after changing seasons?				0.639					
	(3) Does your child usually have difficulty falling asleep and/or easily wake up during sleep?					0.437				
QDC	(4) Is your child prone to a lack of energy?							0.589		
	(5) Is your child not always interested in eating?					0.676				
	(6) Is your child’s complexion too yellowish and dull?					0.72				
	(7) Are your child’s muscles soft and not firm enough?					0.602				
	(8) Is your child prone to catching a cold?				0.59					
	(9) Is your child always very timid?	0.612								
	(10) Does your child tend to sweat with a little activity?	0.409							0.416	
YaDC	(11) Does your child always have cold hands and feet?	0.358								
	(12) Is your child often afraid of the cold or afraid of eating (drinking) cold things?								0.514	
	(13) Is your child prone to diarrhea after catching a cold?								0.521	
	(14) Does your child like hot drinks?								0.617	
YiDC	(15) Does your child frequently experience dry skin and lips?			0.583						
	(16) Does your child often have dry or globular stools?			0.641						
	(17) Is your child impatient and prone to losing his temper?	0.514								
	(18) Does your child have difficulty swallowing or feel uncomfortable when eating dry food?								0.447	
	(19) Is your child easily restless or hyperactive?					0.381				
	(20) Does your child sweat easily when sleeping?								0.351	
PDC	(21) Is your child prone to burnout and not like sports?							0.442		0.419
	(22) Is your child’s belly loose and fat?									0.629
	(23) Does your child like to eat sweet or greasy things?				0.422					
	(24) Is your child’s stool loose/unformed?								0.313	
	(25) Does your child often have phlegm in his throat?				0.457					
	(26) Is your child’s tongue coating thick?				0.586					
DHC	(27) Do your child’s eyes get red easily or produce discharge?			0.475						
	(28) Does your child often urinate yellow?			0.603						
	(29) Does your child always have bad breath?				0.421		0.425			
	(30) Does your child often have perianal eczema or red itching?			0.414						0.481
	(31) Are your child’s lips slightly red?			0.461						
BSC	(32) Does your child have purplish-dark nails (fingers/toes)?						0.741			
	(33) Does your child have petechiae on the lips or tongue?						0.698			
	(34) Is your child prone to blue and purple ecchymosis after bumping or fading slowly after occurrence?				0.416		0.467			
	(35) Is your child’s skin or neck, or at the base of the armpit or thigh dark?						0.551			
QSC	(36) Does your child have a cheerful personality?							0.601		
	(37) Is your child prone to fear or fright?	0.618								
	(38) Does your child always cry when encountering difficulties?	0.546								
	(39) Is your child too sensitive or too concerned about the attitude of others?	0.673								
	(40) Is your child green at the root of his nose (in the middle of his eyebrows)?						0.293			
	(41) Is your child prone to being too excited or nervous?	0.668								
SC	(42) Does your child often sneeze in the morning or after catching a cold?		0.656							
	(43) Does your child often have a stuffy nose and a runny nose when he is not sick? (Over 2 weeks)		0.73							
	(44) Is your child prone to allergies (to medication, food, smell, pollen, or during seasons and climate change)?		0.781							
	(45) Does your child often have a skin rash?		0.606							
	(46) Does your child like rubbing his nose or scratching his skin?		0.63							
	(47) Is your child prone to dark circles under the eyes?		0.429							

Extraction Method: Principal Component Analysis. Rotation Method: Kaiser Normalized Varimax Method.

a The rotation converged in 13 iterations.

## 4 Discussion

The majority of existing assessment tools for preschool children primarily focus on specific domains such as cognitive development, physical growth, motor impairments, and the diagnosis of psychological disorders (19). These tools are generally designed for singular diagnostic purposes, aiming to accurately identify children with particular medical or developmental conditions. However, there has been a notable lack of comprehensive instruments that integrate both physical and mental health assessments, especially for the early detection and prevention of sub-healthy states in children. The scale developed in this study addresses this gap effectively by providing a holistic evaluation framework for preschool children’s overall well-being.

Through the aforementioned steps, this study successfully developed a scale demonstrating high feasibility, sound validity and reliability, and reasonable diagnostic utility. However, several aspects still require further exploration and discussion.

Cronbach’s *α* coefficient is one of the most commonly used indices for assessing the internal consistency of a scale. In general, a Cronbach’s *α* value above 0.80 for the total scale and above 0.60 for subscales is considered acceptable, indicating good internal reliability. Several researchers have noted that both excessive and insufficient numbers of items can influence the stability of Cronbach’s *α* (20). Specifically, a reduction in the number of items tends to lower the *α* coefficient; for scales containing only four items, the α value may sometimes fall below 0.60 or even 0.50 (21). In the second phase of this study, the YaDC and BSC subscales each consisted of four items, with Cronbach’s α coefficients of 0.536 and 0.579, respectively. Considering the limited number of items and their conceptual coherence, these values can still be regarded as acceptable, suggesting that the internal consistency of the subscales remains within a reasonable range.

Structural validity analysis indicated that the cumulative variance contribution rate of the extracted common factors reached 60% (Table 5), suggesting that the overall structural validity of the scale is acceptable. However, the validity indices of individual subscales did not reach ideal levels, primarily due to the unique diagnostic features of Traditional Chinese Medicine (TCM) syndromes—namely, the multidimensional and interrelated nature of symptom clusters. This is a very common situation in medicine, and numerous researchers have demonstrated the rational differentiation between syndromes from perspectives such as intestinal flora distribution (22). Specific item combinations could effectively distinguish between different syndrome patterns (e.g., phlegm-dampness vs. damp-heat constitutions, as shown in Table 6). Symptom overlap among syndromes led to cross-loading issues within the subscale factor structures. For example, due to the presence of “dampness” factors, both phlegm-dampness and dampness-heat types easily manifest as sticky stools. This issue has also been reported in a previous study (13, 15). Although earlier analyses, including discrete trend analysis, homogeneity testing, and item-total correlation (coefficients > 0.4), demonstrated acceptable item discriminative validity, the polysemous nature of core symptoms—often key to clinical differentiation—inevitably contributes to increased structural complexity.

In the development of questionnaires or rating scales, statistical analysis is typically regarded as a key foundation, as it directly influences the scale’s reliability, validity, and structural modeling. Structural validity is considered particularly crucial. At present, psychometric evaluation of structural validity primarily relies on confirmatory factor analysis (CFA), or a combination of exploratory factor analysis (EFA) followed by CFA. Moreover, CFA is based on hypothetical models (such as single factor, multi factor, second-order factor), requiring that the relationships between items highly conform to statistical patterns (23). However, as mentioned above, clinical symptoms are often multidimensional, intersecting, and dynamic, making it difficult to fully explain by a fixed factor model. This method often encounters model-fitting issues in the development and validation of clinical and psychological scales. Several scales that are currently considered the “gold standard” in psychiatry, such as the Hamilton Depression Scale, have encountered these problems. However, the poor CFA results did not affect the clinical efficacy (24).

Therefore, considering that the confirmatory factor analysis (CFA) conducted in this study yielded unsatisfactory model fit indices, and in view of the methodological limitations of further CFA-based exploration as well as the complexity of analyzing constitution-related items in children, exploratory factor analysis (principal component analysis) was ultimately adopted as the primary analytical approach. This method allowed for a more comprehensive examination of the underlying factor structure. The analysis revealed classification deviations in certain constitution-related items, which may be attributed to pediatric-specific factors. First, constitution-sensitive indicators validated in adult scales—such as typical symptoms of Yang deficiency—tend to be less pronounced in children. Second, several pediatric-specific items exhibited cross-loading across multiple constitution types during factor analysis, likely due to the unique physiological characteristics of children. Through expert consensus, these items were designated as auxiliary diagnostic items. In expert consultations, TCM pediatric specialists also noted that the prevalence of Yin deficiency in children is relatively low, which may explain the consistently low factor loadings for related items.

In this study, expert opinions received more attention and application. We tried to make the experts see the original items, and the set of expert references to these original situations quickly concentrated the expert advice. Under the advice of experts, we tried to do further clinical research on the items with TCM particularity or characteristics, or what experts thought was more important or meaningful, even if these items did not perform well in the statistical analysis:

- Item 16 in Table 3—“Does your child like hot drinks?”—is rooted in the TCM pathophysiological concept of “Yang deficiency leading to internal cold.” Although experts unanimously agreed (100% agreement) that the item effectively captures the diagnostic feature of “aversion to cold and preference for warmth,” there were concerns about how clearly parents could understand and interpret this item (Kendall’s W = 0.62). After two rounds of clinical testing and iterative revision of the item’s wording, principal component analysis revealed a factor loading of 0.429. The item was significantly correlated with the total score of the Yang-deficiency subscale (*r* = 0.53, *p* < 0.01) and demonstrated acceptable internal consistency (Cronbach’s *α* = 0.71). These empirical findings support the theoretical validity of cold–heat differentiation in TCM. Therefore, the item was retained, with the hope that it may also be of reference value to other researchers working on pediatric constitution assessment.
- Item 15 in Table 3 – “Does your child often wet the bed?” – was developed based on the TCM theory of “kidney yang deficiency leading to failure of bladder control.” Although supported by 76% of Delphi panel experts, factor analysis showed cross-loading across multiple constitution types: the factor loading for Yang deficiency was moderate and significant (*λ* = 0.549), while the loadings for Qi stagnation (*λ* = 0.333) and damp-heat constitution (*λ* = 0.357) were low. In the forced extraction of nine factors, the item even demonstrated a negative loading under the damp-heat constitution (*λ* = −0.382), suggesting structural inconsistency. A subgroup analysis of children aged 3–6 years with enuresis (*n* = 127) revealed that the most prevalent constitution types were Yin deficiency (38.6%) and Qi stagnation (29.1%), which notably differ from prior clinical findings that predominantly associate enuresis with Yang deficiency (25).

This divergence may be attributed to two key factors:

Differences in study populations: Our research was based on large-scale community screening in kindergartens, while existing clinical studies primarily sampled children who sought medical treatment. These clinical subjects may represent more severe cases of enuresis. In contrast, our sample may include children with milder symptoms, possibly already relieved by early intervention. Notably, in our factor analysis, Yang deficiency remained the only constitution type with a factor loading above the conventional threshold (*λ* > 0.4), suggesting some level of theoretical and structural consistency.

Influence of emotional factors: According to both modern psychological studies and Traditional Chinese Medicine (TCM) theory, emotional dysregulation can contribute to the development of enuresis. Structural equation modeling (SEM) in our study demonstrated a significant association between enuresis symptoms and scores on a pediatric anxiety scale (*β* = 0.41, *p* = 0.003), supporting the emerging concept of “emotional enuresis” (26). This finding aligns with TCM perspectives, wherein Yin deficiency often manifests as irritability, restlessness, and internal heat, while Qi stagnation is associated with introversion, emotional repression, and heightened sensitivity. In our factor analysis, enuresis showed meaningful loadings on items related to Yin deficiency, dampness, and Qi stagnation—patterns that may underlie functional or stress-induced enuresis in preschool children.

Although Item 15 passed content validation by TCM experts and retained some empirical support (i.e., its association with Yang deficiency), its overall discriminative power was relatively low (Youden index = 0.31, significantly lower than that of other items). Considering the unique characteristics of the kindergarten assessment environment—including potential parental reporting bias—the item was ultimately removed from the general pediatric scale. However, due to its theoretical value and potential clinical applicability, we recommend retaining this item in the specialized version of the scale intended for use by trained TCM practitioners in medical or hospital settings.

Although this study has not yet fully verified the model’s robustness through confirmatory factor analysis (CFA) and cross-validation, the exploratory factor analysis (EFA) results demonstrated strong theoretical and clinical alignment with the core concepts of traditional Chinese medicine (TCM) syndromes. This indicates that the extracted nine-factor model possesses good theoretical interpretability and practical potential at this stage. Due to the limitations of sample size and the exploratory nature of this research phase, further investigation into the factor structure could not be conducted. Future studies will employ larger and independent samples to perform CFA and cross-validation, thereby enhancing the verification of the stability and generalizability of the proposed nine-factor model. Such efforts will contribute to the refinement of the pediatric constitution scale and the advancement of theoretical understanding in this field.

### 4.1 Strengths and limitations

The development and optimization of scale items integrated expert consensus with statistical validation, taking into account multidimensional influencing factors such as children’s psychology, lifestyle habits, behavioral characteristics, and developmental status. The scale combines subjective perceptions with objective symptoms, requires a short measurement time, and is designed for completion by parents/caregivers, making it suitable for general population application.

This study has several limitations. First, it is a preliminary investigation. Participants for psychometric analysis were recruited from schools, which may limit the generalizability of the scale. Future studies should involve larger-scale and more rigorous designs. Second, this study did not include criterion validity analysis, mainly because research on traditional Chinese medicine (TCM) constitutions in children aged 3–6 years remains limited. At present, no widely recognized and validated instrument in China can serve as a “gold standard” for comparative validation. Therefore, criterion-related validity analysis could not be conducted in this study. Future research will consider using expert-based clinical judgments as a reference to compare with the scale assessment results in order to further examine and enhance the validity and practical applicability of this instrument. Third, while the original CCMQ has been translated into multiple languages and widely used globally, the cross-cultural applicability of the pediatric CCMQ requires further validation. Finally, in the preliminary analysis phase, we attempted confirmatory factor analysis (CFA), but it did not pass statistical tests. Therefore, only exploratory factor analysis (EFA) was conducted for structural validity analysis, and no adequate statistical support was obtained. As a result, while the psychometric properties of the current scale are still acceptable, there are limitations in the scale development method based on Traditional Chinese Medicine (TCM), particularly due to the long-standing structural contradictions related to the principle of syndrome differentiation in TCM. Future studies could attempt sample cross-validation based on larger-scale research while gradually improving the development and validation system for TCM-specific statistical methods. We also suggest increasing the involvement of statistical experts in this interdisciplinary research.

## 5 Conclusion

This study developed and evaluated a 47-item pediatric health assessment questionnaire called the Wang’s TCM Constitution Questionnaire (3–6 years). It successfully retains the original nine constitution-type structure from the CCMQ, demonstrates acceptable reliability and validity through psychometric evaluation, and can be widely applied to 3–6-year-old Chinese preschoolers. This tool provides a simple and practical questionnaire for large-scale screening of children’s TCM constitutions, supporting primary healthcare and health management.

## Data Availability

The data analyzed in this study is subject to the following licenses/restrictions: the original contributions presented in the study are included in the article/supplementary material, further inquiries can be directed to the corresponding author. Requests to access these datasets should be directed to liwenle1992@qq.com.

## References

[1] Loveday S White N Constable L Gates A Sanci L Goldfeld S . Lessons learned from the implementation of an integrated health and social care child and family hub - a case study. Int J Integr Care. (2024) 24:9. doi: 10.5334/ijic.8631, PMID: 39552790 PMC11568806

[2] Ortega FB Ruiz JR Castillo MJ Sjöström M. Physical fitness in childhood and adolescence: a powerful marker of health. Int J Obes. (2008) 32:1–11. doi: 10.1038/sj.ijo.0803774, PMID: 18043605

[3] Haav A Oja L Piksööt J. The influence of kindergarten environment on the development of preschool children’s physical fitness. Int J Environ Res Public Health. (2024) 21:761. doi: 10.3390/ijerph21060761, PMID: 38929007 PMC11203701

[4] Wang J Yu RX Wang Q. New concept of health with perspective of Chinese medicine. Chin J Integr Med. (2019) 25:712–5. doi: 10.1007/s11655-016-2671-2, PMID: 27107573

[5] Wang J Wang T Shuai LY Fei ZY Ru LL Wang Q. Research on constitution of Chinese medicine and implementation of translational medicine. Chin J Integr Med. (2015) 21:389–93. doi: 10.1007/s11655-014-2019-8, PMID: 25519443

[6] Wang Q. Chinese medicine constitution theory. China: People’s Health Publishing House (2008).

[7] Sun W Bai M Wang J Wang B Liu Y Wang Q . Machine learning-assisted rapid determination for traditional Chinese medicine constitution. Chin Med. (2024) 19:127. doi: 10.1186/s13020-024-00992-0, PMID: 39278905 PMC11403957

[8] Jing H. The development of constitution questionnaire of Chinese medicine in English edition and the epidemiological investigation of TCM constitution in American and Canadian population. Beijing: Beijing University of Chinese Medicine (2012).

[9] Li B Cao H Tian E Wang Q. A cross-sectional study of a Korean population using the standardized constitution in Chinese medicine questionnaire (Korean version). J Anhui Univ Chin Med. (2015) 34:25–8. Available online at: https://kns.cnki.net/kcms2/article/abstract?v=IPzr95zWmwThQujCAzZfGsVPXIPRBA4md26w1fJiMJUX4_IOOq476ANgpQmBjX6527kuXsd6VjUUXMJ8FO8VJ373kOfB0yzqjdah3ug3zVoahsCEG4hQn4i3Z_Q46JJstGeF95b5wKx2r65Uuj8ldLtM63ObQy7zygKciHdYRCpFTlDjBa6wc9MiU8sNWEu-&uniplatform=NZKPT&language=CHS

[10] Lai Y. Network survey research and result analysis of body constitution of TCM among overseas population. Beijing: Beijing University of Chinese Medicine (2014).

[11] Li Y. Research progress of children’s constitution of traditional Chinese medicine. China Modern Dist Educ Tradit Chin Med. (2024) 22:165–8. Available online at: https://kns.cnki.net/kcms2/article/abstract?v=IPzr95zWmwT3PcG7EqW-ktbkuz8T-VpziVFw9AprcjJ8s5_XUN7aYQ1YRRnxa20-SBA5ipGsGmssUZZ_hjKEaD6itwH73SgI-Fu1QbEbQl9TIR6qHcbu9yh0oSpoDqatW0Uv30uVAGIRtnDHvLCvhkl31DVwLuWgOVguq0q9s0DKJL0nq14fDKro3Pql2S4z&uniplatform=NZKPT&language=CHS

[12] Cao H Chen X Song Y Li SX Ma H Zhang G . A comprehensive study of psychological well-being and traditional Chinese medicine constitutions among model workers in Beijing. Front Psych. (2024) 15:1425757. doi: 10.3389/fpsyt.2024.1425757, PMID: 39323969 PMC11422221

[13] Li Z. Development and evaluation of Wang’s nine TCM constitution questionnaire (0–3 version). Beijing: Beijing University of Chinese Medicine (2021).

[14] Yang Y. Development of TCM constitution questionnaire for children aged 7–14 years. Beijing: Beijing University of Chinese Medicine (2015).

[15] Bai M Li Z Wang HY Ma XL Wang ZL Li SJ . Development and evaluation of short-form version of the constitution in Chinese medicine questionnaire: study a new and best brief instrument of Chinese medicine for health management. Chin Med. (2023) 18:140. doi: 10.1186/s13020-023-00844-3, PMID: 37904166 PMC10617149

[16] Zeng G. Modern epidemiological methods and applications. Beijing: Joint Publishing House of Beijing Medical University and Peking Union Medical College (1994).

[17] Wang Y. Development and evaluation of medical scales: theories, methods, and case studies. Beijing: Peking University Medical Press (2020).

[18] Wu M. Questionnaire statistical analysis practice: SPSS operation and application. Chongqing: Chongqing University Press (2010).

[19] Yang Y. Child developmental behavioral and psychological assessment scales. Beijing: People’s Medical Publishing House (2016).

[20] Zhang L. The positive and misuse of reliability. J Beijing Sport Univ. (2002) 3:348–50.

[21] Jiang X Shen Z Zhang N Liao H Xu H. Reliability and validity analysis of questionnaire. Modern Prev Med. (2010) 37:429–31. Available online at: https://kns.cnki.net/kcms2/article/abstract?v=IPzr95zWmwSeuNt3gO60ZL7AzdDJj4uI0kYGta1DGmyP6dm8Z3ewBbXqpsSTGB4VygHmGe1Ve6vNsMGOmh22xbcBQjJK8vViKhSYVHwy67k-7SYRy1q9TAQRiqv9Q2pgNWx3618dztk2WKhESAwsH1KrI_8aLu2u1Yn7uGM-9vtvoEc8-Jk6KQiFiaJudHiC&uniplatform=NZKPT&language=CHS

[22] Yuan S Zhang R Zhu Z Zhou X Zhang H Li X . Potential metabolic markers in the tongue coating of chronic gastritis patients for distinguishing between cold dampness pattern and damp heat pattern in traditional Chinese medicine diagnosis. J Inflamm Res. (2025) 18:9717–34. doi: 10.2147/JIR.S480405 40718131 PMC12296634

[23] Richardson GB Bates DG McLaughlin LE McGee N Tse WWY Lai MHC. Are higher-order constructs in evolutionary psychology attributable to omitted cross-loading bias? An exploratory structural equation modeling approach. Hum Nat. (2025) 36:257–80. doi: 10.1007/s12110-025-09497-7 40694251 PMC12417301

[24] Cole JC Motivala SJ Dang J Lucko A Lang N Levin MJ . Structural validation of the Hamilton depression rating scale. J Psychopathol Behav Assess. (2004) 26:241–54. doi: 10.1023/B:JOBA.0000045340.38371.04

[25] Tao M. Correlation between TCM syndrome types and constitution in children with enuresis and clinical study on TCM treatment. Zhejiang: Zhejiang Chinese Medical University (2014).

[26] Gao L. Clinical research progress on pediatric enuresis. Modern Med Health Res Electr J. (2023) 7:138–41. Available online at: https://kns.cnki.net/kcms2/article/abstract?v=IPzr95zWmwT2nU-jy-Rj0vDigg9XCYNySYCt2HU35TisjHz_YWbw1wL4ufUu2dQG9zooNNB-roHN7aixT1ta45gjHDmbi49KieIRSboSAADRXz39firIfBvwgQ_x_QEEAO6UqRkpPl02EcIw4NAFzgMIslT61Bxceq0OvYNZgQLJNAhJPhtSRwEg1Qve66nS&uniplatform=NZKPT&language=CHS

